# Characterization of membrane penetration and cytotoxicity of C9orf72-encoding arginine-rich dipeptides

**DOI:** 10.1038/s41598-018-31096-z

**Published:** 2018-08-24

**Authors:** Kohsuke Kanekura, Yuichiro Harada, Mao Fujimoto, Takuya Yagi, Yuhei Hayamizu, Kentaro Nagaoka, Masahiko Kuroda

**Affiliations:** 1 Department of Molecular Pathology, Tokyo Medical University, Tokyo, Japan; 2 School of Life Sciences, Tokyo University of Pharmacy and Life Sciences, Tokyo, Japan; 3 Department of Neurology, KEIO University, Tokyo, Japan; 4 Department of Organic and Polymeric Materials, Tokyo Institute of Technology, Tokyo, Japan; 5 Laboratory of Veterinary Physiology, Department of Veterinary Medicine, Tokyo University of Agriculture and Technology, Tokyo, Japan

## Abstract

Cell-penetrating peptides (CPPs) including arginine-rich peptides are attracting a lot of attention due to their potential as a novel intracellular drug delivery tool without substantial toxicity. On the other hand, disease-associated arginine-rich CPPs, such as poly-PR and poly-GR translated from C9orf72 gene, also efficiently enter neuronal cells and then kill them. Although both non-harmful CPPs and harmful poly-PR/GR penetrate the plasma membrane using same arginine residues, little is known about the factors which determine the toxicity of the pathogenic CPPs. Here, we show that poly-PR and poly-GR, but not other Arg-rich CPPs, specifically distributed to nucleolus via interaction with RNA. Importantly, C9orf72-dipeptides, but not other Arg-rich CPPs, caused inhibition of protein translation and cell death. Raising extracellular pH enhanced the cell penetration of poly-PR. The repeat number of (PR) affected the secondary structure and determined the intracellular delivery rate and neurotoxicity, and enforced intracellular delivery of non-penetrating short poly-PR peptide caused cell death, suggesting that modulation of extracellular environment to inhibit the uptake of Arg-rich dipeptides might be a drug target against poly-PR/GR-mediated neurotoxicity.

## Introduction

Dipeptide-repeat proteins (DRPs), translated through repeat-associated non-ATG (RAN) translation from pathogenic G4C2 expansions in C9orf72 gene, are supposed to play a pivotal role in development of C9orf72-related neurological disorders such as amyotrophic lateral sclerosis (ALS) and frontotemporal dementia (FTD)^1–4^. Among 5 species of DRPs (poly-GA, GP, PR, GR and PA) translated from sense and antisense of G4C2 expansions, especially arginine-rich DRPs (poly-PR and poly-GR) are of interest because they caused neuronal loss *in vitro* and *in vivo*^3,4^. When these arginine-rich DRPs are applied to the culture medium of neuronal cells, they penetrate the plasma membrane and directly affect intracellular machineries, thus they are classified as cell-penetrating peptides (CPPs)^4,5^.

**Figure 1:**
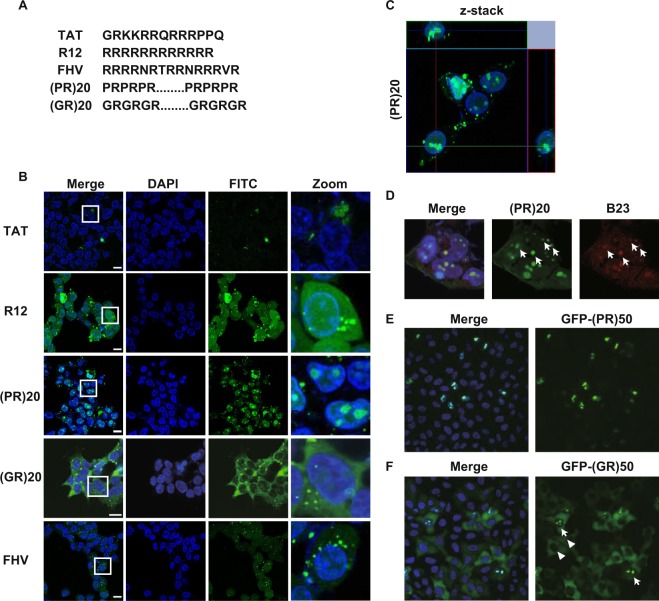
Poly-PR localized to nucleoli. (**A**) Amino acid sequences of cell penetrating peptides (CPPs) used in this study. (**B**) Confocal imaging of live HEK293 cells treated with 10 μM of indicated FITC-labeled CPPs. Nucleus was visualized by Nucblue live dye. (**C**) Z-stack imaging of live HEK293 cells treated with FITC-(PR)20. (**D**) Immunocytochemistry of HEK293 cells treated with FITC-(PR)20, immunostained with anti-B23/nucleophosmin antibody. Nucleus was visualized by DAPI. (**E**) Hela cells were transfected with GFP-(PR)50 repeats. (**F**) Hela cells were transfected with GFP-(GR)50 repeats. White arrows show the cells with both nucleolar and cytosolic staining and white arrowheads indicate the cells with cytosolic staining. Scale bar shows 20 μm.

The first CPP discovered was trans-activating transcription activator (TAT) peptide from human immunodeficiency virus-1 (HIV-1) whose amino acid sequence is shown in Fig. 1A ^6^. Since the discovery, a variety of CPPs have been artificially designed (e.g. R8, R12) or identified from natural organisms such as Flock house virus (FHV)^7^. Because of their efficient translocation through biological membranes without significant cellular damage, many of them have been extensively investigated as tools for intracellular delivery of compounds including nucleic acids, proteins and drugs^8–10^. Although several hypotheses such as the endocytotic pathway, energy-independent uptake and cell surface molecules-mediated uptake have been proposed, precise mechanisms how these CPPs penetrate the plasma membrane still remain to be investigated^11^.

Similar to these non-harmful CPPs, ALS-causing poly-PR/GR efficiently enter cells^4,5^. Once they penetrate the plasma membrane, intracellular poly-PR/GR localize to membrane-less organelles and inhibit their functions, resulting in disturbance of protein translation, abnormal formation and malfunction of stress granules, mis-splicing of mRNAs, and ultimately neuronal death *in vitro* and *in vivo*^3–5,12,13^. Despite their importance in neuroscience and drug delivery research fields, the mechanism underlying the membrane penetration as well as the correlation between membrane penetration and neurotoxicity of C9orf72-DRPs remains undetermined.

Here we identified key factors which affect membrane penetration and neurotoxicity by poly-PR/GR peptides. First, we examined a series of arginine-rich CPPs and found that poly-PR and poly-GR, but not other CPPs, distributed to nucleolus. Next, *in vitro* translation assay and lactate dehydrogenase (LDH) release assay showed that C9orf72-DRPs, but no other CPPs, inhibited protein translation and caused cell death by aberrant interaction with RNA. The cell penetration rate of poly-PR was determined by length of repeats and extracellular pH, and enforced delivery of non-penetrating short poly-PR peptide caused cell death, suggesting that inhibition of intracellular delivery of DRPs might be a novel drug target for C9orf72-mediated ALS.

## Results

Cell penetrating peptides (CPP) which can cross the plasma membrane bilayer are subcategorized by their biochemical characteristics such as hydrophilic CPPs, hydrophobic CPPs and amphipathic CPPs^14,15^. C9orf72-related Arg-rich dipeptides are classified as the hydrophilic cationic CPP because 50% of their content is polar arginine. The most established cationic CPP is TAT peptide (GRKKRRQRRRPPQ: 13 a. a.) identified from HIV-1. Since the discovery of TAT, many cationic CPPs including R12 (RRRRRRRRRRRR: 12 a. a.) and FHV peptide (RRRRNRTRRNRRRVR:15 a. a.) were identified and investigated as possible tools for the drug delivery system^16^. The advantage of these CPPs as a drug delivery carrier is their high ability to deliver molecules including proteins and nucleotides across biological membranes without cytotoxicity. Although C9orf72-encoding Arg-rich dipeptides should have similar biochemical characteristics with these CPPs, C9orf72-related DRPs have been shown to cause cell death once they penetrate the plasma membrane^4^.

To clarify the difference between the C9orf72-related DRPs and non-harmful CPPs, we synthesized well-known cationic CPPs including TAT peptide, R12 peptide and FHV peptide and C9orf72-derived DRPs, (PR)20 peptide (40 a. a.) and (GR)20 peptide (40 a. a.) (Fig. 1A), and examined their penetration to human embryonic kidney (HEK) 293 cells. All of the CPPs examined were uptaken by HEK293 cells as expected (Fig. 1B). The confocal imaging revealed the subcellular localization of FITC-labeled CPPs. TAT made cytosolic large dots, R12 and FHV diffusely localized to both nuclear and cytosol with some punctate structures in cytosol, (PR)20 mainly localized to the nucleus and especially accumulated to round-shaped nucleoli and (GR)20 localized to both nucleoli and cytosol (Fig. 1B). The nucleolar localization of (PR)20 was also confirmed with Z-stack imaging and colocalization with a nucleolar marker B23/nucleophosmin (Fig. 1C,D) as reported previously^4,12^. To deny the possibility that nucleolar distribution of poly-PR and poly-GR was an artifact because these peptides were added extracellularly, we overexpressed green fluorescent protein (GFP)-tagged (PR)50 repeat protein and GFP-tagged (GR)50 repeat protein in HEK293 cells and confirmed that both GFP-(PR)50 and GFP-(GR)50 distributed to nucleoli (Fig. 1E,F). Consistent with the subcellular localization of FITC-peptides, GFP-(PR)50 showed almost exclusive distribution to nucleoli and GFP-(GR)50 distributed to both nucleoli and cytosol (Fig. 1E,F).

**Figure 2:**
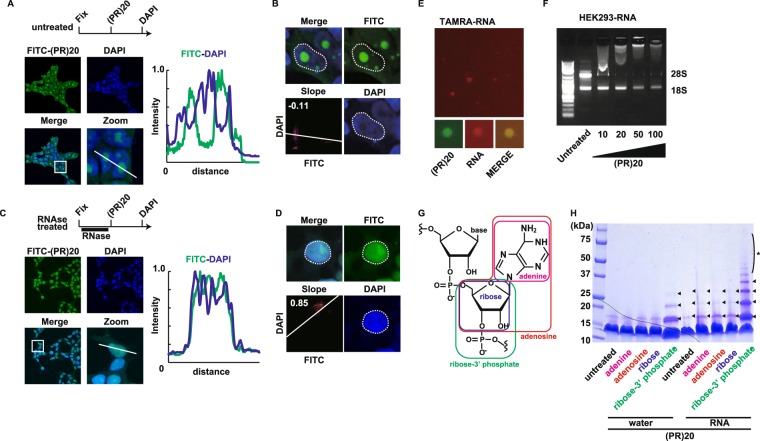
Poly-PR interacts with RNA. (**A**) Confocal imaging of HEK293 cells fixed/permeabilized and stained with FITC-(PR)20 in the absence of pretreatment of RNase A. (**B**) Colocalization analysis of signals from DAPI staining and FITC-(PR)20 in the HEK293 cells in the absence of pretreatment of RNase A. (**C**) Confocal imaging of HEK293 cells fixed/permeabilized and stained with FITC-(PR)20 in the presence of pretreatment of RNase A. (**D**) Colocalization analysis of signals from DAPI staining and FITC-(PR)20 in the HEK293 cells in the presence of pretreatment of RNase A. (**E**) Liquid-liquid phase separation of tetramethylrhodamine (TAMRA)-labeled random 15-mer RNA by FITC-(PR)20. (**F**) Agarose gel electrophoresis of human ribosomal RNA mixed with (PR)20 peptide at indicated concentration (μM). (**G**) Structure of RNA (base: adenine). (**H**) Crosslinking analysis of HA-(PR)20 peptide mixed with adenine, adenosine, ribose or ribose-phosphate in the presence or absence of RNA (poly-A). Arrowheads indicate crosslinked oligomer of HA-(PR)20 peptides. *polymerized HA-(PR)20.

The nucleolar distribution of the FITC-labeled DRPs and GFP-fused DRPs implies existence of molecules with high affinity to C9orf72-DRPs in the nucleolus. The nucleolus is a subnuclear membrane-less compartment which is the site for transcription of ribosomal RNA and assembly of ribosome^17^. Because the nucleolus contains a large amount of ribosomal RNA accounting for 90% of total RNA and a number of RNA-binding proteins, the concentration of intranucleolar DNA is low, since DNA staining by DAPI excludes the nucleolus^17^. In our previous study, we demonstrated liquid chromatography mass spectrometry to comprehensively identify interactors of poly-PR and found that poly-PR interacted with RNA-binding proteins. Immunoprecipitation assays in the presence of RNAse diminished some of the interactions, suggesting that poly-PR directly binds to RNA^5^. In order to examine whether (PR)20 accumulated to the nucleoli through interaction with RNA or with RNA-binding proteins, we fixed the HEK293 cells with 4% paraformaldehyde (PFA) and incubated the cells with FITC-(PR)20 with or without RNAse treatment (Fig. 2A–D). Fixation of HEK293 cells slightly increased cytosolic staining by FITC-(PR)20 compared with the staining of live HEK293 cells (Fig. 1B) but most of (PR)20 localized to nucleoli (Fig. 2A). The RNAse treatment diminished the specific distribution of (PR)20 to nucleoli and increased diffuse nuclear staining (Fig. 2C). Colocalization analysis showed that the green fluorescence from FITC-(PR)20 and blue fluorescence from DAPI did not correlate before the RNAse treatment (slope m = −0.11), and the RNAse treatment drastically change the correlation manner (slope m = 0.85), implying that (PR)20 distributed to nucleoli through interaction with RNA, presumably ribosomal RNA, abundant in the nucleolus (Fig. 2B,D).

We next investigated how (PR)20 interacts with RNA. It has been reported that poly-PR forms phase-separated liquid droplets with anionic molecules *in vitro*^12,13^. Therefore, we first tested if (PR)20 induces phase-separated liquid droplets with RNA. For this purpose, we synthesized tetramethylrhodamine (TAMRA)-labeled random 15-mer RNA and mixed the RNA with FITC-labeled (PR)20. As expected, (PR)20 induced the round-shaped droplet formation (Fig. 2E), indicating that (PR)20 induced phase separation of RNA. We also confirmed that (PR)20 induced separation of dense liquid phase fusing each other, and the separated droplets showed high circularity and roundness, suggesting minimization of surface tension (Figs S1A and 1B). To examine whether (PR)20-RNA complex maintains the liquid-like aspect in live cells, we demonstrated the fluorescence recovery after photobleaching (FRAP) assay and confirmed that the nucleolar (PR)20 peptide maintained fluidity even after 24 hr incubation (Fig. S1C,D). Next, we tested if (PR)20 interacts with ribosomal RNA. The ribosomal RNA is negatively charged and runs to anode when applied to Tris-acetate-EDTA (TAE)-buffered agarose gel electrophoresis. (PR)20 is positively charged and thus possibly neutralizes the negative charge of ribosomal RNA, inhibiting the running during the electrophoresis. To test if this hypothesis is the case, we mixed the human ribosomal RNA extracted from HEK293 cells and (PR)20 peptide then applied the mixture to the agarose gel electrophoresis. As expected, (PR)20 inhibited the running of rRNA to the anode and the rRNA resided to the well applied (Fig. 2F). To support this, we next tested which component of RNA is the binding target for poly-(PR) peptide. For this end, we used a crosslinking assay because RNA enhances polymerization of poly-(PR)^5^. RNA is composed of nucleotides containing bases, sugars and phosphates (Fig. 2G), and each component was mixed with (PR)20 in the presence or absence of poly-A RNA as an enhancer for polymerization. Ribose-3′ phosphate, but not ribose alone, enhanced oligomerization of (PR)20, suggested that the phosphate attracted (PR)20 peptide presumably due to the negative charge (Fig. 2H).

**Figure 3:**
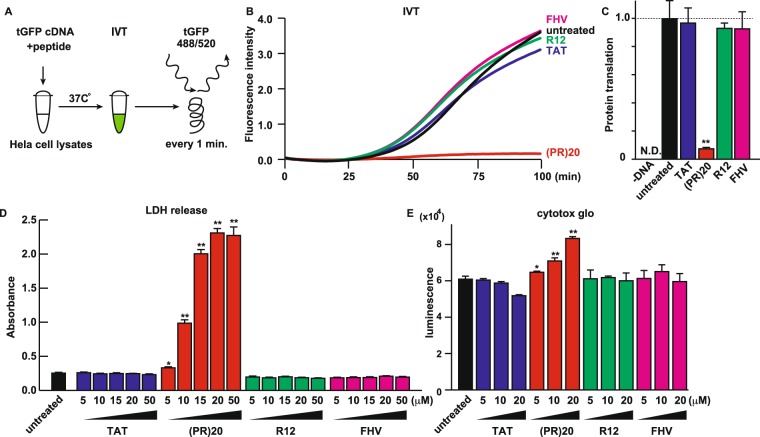
Poly-PR caused cell death through inhibition of protein translation. (**A**) Scheme of real-time monitoring of protein translation using *in vitro* translation (IVT) system. tGFP: turboGFP. (**B**) Real time monitoring of tGFP signal in the IVT assay. Signals from tGFP were measured every 1 min. (**C**) Fluorescence intensity of translated tGFP in the IVT assay treated with 100 μM of each peptide. N = 3. (**D**) Cytotoxicity of each CPPs were monitored by release of LDH. NSC34 cells were treated with each CPP at indicated concentration for 24 hr. N = 6. (**E**) Cytotoxicity of each CPPs were monitored by Celltox glo luminescence. NSC34 cells were treated with each CPP at indicated concentration for 24 hr. N = 6. Each experiment was independently repeated at least 3 times. Asterisks indicate a significant difference analyzed by one-way ANOVA followed by Dunnett’s test (**p < 0.01, *p < 0.05).

As we previously reported, Arg-rich C9orf72 dipeptides inhibit protein translation and cause neuronal cell death^5^. Indeed, ectopic expression of GFP-(PR)50 and GFP-(GR)50 inhibited protein translation in Hela cells (Fig. S2A). To investigate if other cationic CPPs affect protein translation and exert neurotoxicity as well, we tested the effects of the cationic CPPs on protein translation. For this end, we adopted a cell-free *in vitro* translation (IVT) system to avoid possible secondary effects of cytotoxicity by the CPPs (Fig. 3A). The efficiency of protein translation was real-timely monitored by fluorescence emitted from translated turboGFP which does not have significant delay for maturation like jellyfish-derived GFP^18^. As expected, (PR)20 (Arg content: 50%), but not TAT (Arg content: 46%), R12 (Arg content: 100%) or FHV (Arg content: 73%), efficiently inhibited protein translation *in vitro* (Fig. 3B,C). Next, motor neuronal NSC34 cells were treated with these CPPs and cellular damages were measured by LDH release assay and Cytotox glo assay (Fig. 3D,E). Consistent with the results of IVT assay, (PR)20, but not other CPPs, killed the cells, indicating that not only the ratio of cationic amino acid content but structural factors underlie the cell death by poly-PR.

**Figure 4:**
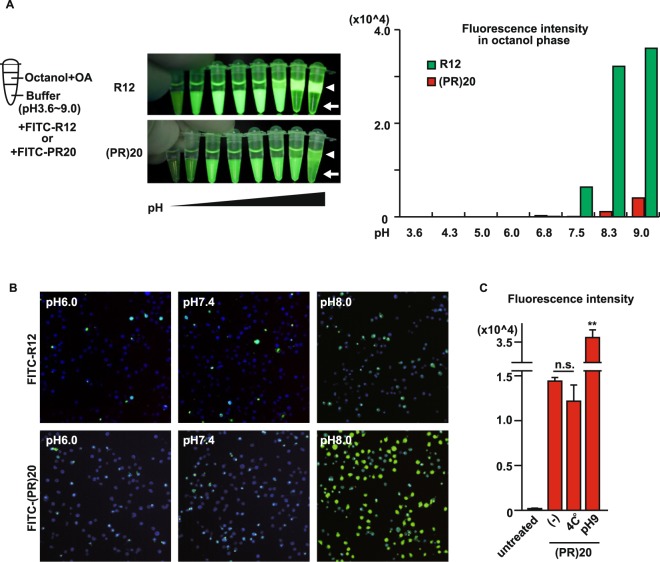
Extracellular pH affected penetration of poly-PR. (**A**) Peptides absorption assay using FITC-R12 or FITC-(PR)20. The pictures show the actual images of the absorbed peptides. The white arrowheads indicate octanol phase and white arrows show aqueous buffer phase. The bar graph shows the fluorescence of FITC-labeled peptides absorbed in the octanol fractions measured by a plate reader. (**B**) Hela cells were treated with PBS with different pH containing 2 μM FITC-R12 or FITC-(PR)20. Nucleus was visualized by Nucblue live dye. (**C**) Cell penetration rate of FITC-(PR)20 incubated at 37°C or 4°C or pH9 in HEK293 cells, measured by a plate reader. Asterisks indicate a significant difference analyzed by one-way ANOVA followed by Dunnett’s test (**p < 0.01, *p < 0.05). n.s.; not significant.

Next, we investigated the molecular mechanism how poly-PR penetrates the plasma membrane. It has been shown that cationic CPPs cross the plasma membrane via both the energy-independent translocation pathway and energy-dependent endocytotic pathway. To confirm if this is the case with poly-PR, we first tested the effect of fatty acids and pH gradient at the cell membrane on the translocation of the (PR)20 peptide, because these two components were reported to affect the uptake of Arg-rich peptides by live cells through the energy-independent translocation^19^. To see the effects of fatty acids and pH gradient on the translocation of the peptides, we adopted a simple cell free model to show the distribution of fluorescent CPPs between an aqueous buffer and hydrophobic octanol containing oleic acid, mimicking lipid bilayer environment^19^. When pH was increased higher than 7.5, R12 started to be absorbed into the octanol phase and most of R12 peptide was absorbed into octanol phase at pH 9.0 (Fig. 4A, upper panels). The pH gradient also enhanced the absorption of (PR)20 peptide into the octanol phase and higher pH increased the absorption of the peptide (Fig. 4A, lower panels), indicating that extracellular pH condition may influence the uptake of Arg-rich dipeptides in hydrophobic environments such as the lipid bilayer. The change of pH did not enhance the absorption of non-permeable human insulin peptide into the octanol phase (Fig. S2B), indicating that the effect of pH was specific to the cationic CPPs. Next, we challenged Hela cells with buffers of different pH and treated with FITC-R12 or FITC-(PR)20 (Fig. 4B). Consistent with the results from the cell-free model (Fig. 4A) and previous study^19^, higher pH increased the distribution of FITC-labeled CPPs into the cells. We also investigated whether cellular uptake of (PR)20 is affected by inhibition of energy-dependent endocytosis. It has been established that the endocytotic pathway is temperature-sensitive^16^, thus we tested if lowering temperature affects the uptake of the peptide. When the cells were incubated with FITC-(PR)20 at 4 °C, the fluorescent signal was comparable with the signal from the cells incubated at 37 °C (Fig. 4C). These results suggest that (PR)20 penetrated the plasma membrane via the direct translocation rather than the energy-dependent endocytosis.

**Figure 5:**
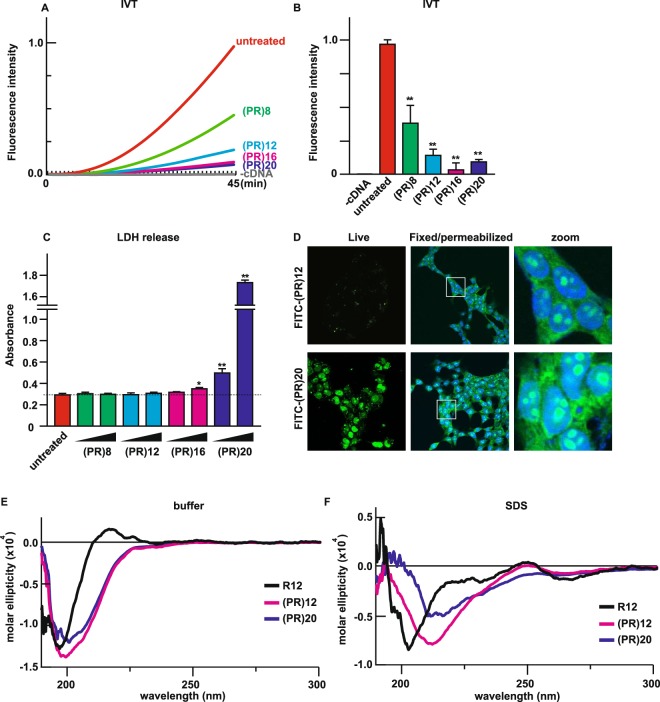
Repeat lengths affected the cytotoxicity of poly-PR peptides. (**A**,**B**) Fluorescence intensity of translated tGFP in the IVT assay treated with 100 μM of each peptide. N = 3. (**C**) Cytotoxicity of poly-PR peptides with different repeat lengths were monitored by release of LDH. NSC34 cells were treated with each peptide at 5 μM or 10 μM for 24 hr. N = 6. (**D**) Confocal imaging of HEK293 cells stained with 10 μM of FITC-(PR)12 or FITC-(PR)20 before or after the fixation/permeabilization step. (**E**) CD spectra of R12, (PR)12 and (PR)20 in Tris-buffer (pH7.4). (**F**) CD spectra of R12, (PR)12 and (PR)20 in 1 v/w% SDS-Tris buffer (pH7.4). Asterisks indicate a significant difference analyzed by one-way ANOVA followed by Dunnett’s test (**p < 0.01, *p < 0.05).

Next, we investigated the effect of repeat length of PR on the cell toxicity. IVT assay indicated that even (PR)8 blocked the protein synthesis and longer repeat tended to have higher inhibition rate (Fig. 5A,B). We also examined the effect of repeat length on the poly-GR toxicity. We performed the IVT assay with poly-GR peptides with 12 repeats and 20 repeats. In this assay, amyloid β (40 a.a.) was used as a negative control peptide with length of 40 a. a., same as (GR)20 (Fig. S3A,B). (GR)20 modestly inhibited protein translation and we did not see inhibitory effect of (GR)12 or amyloid β on protein translation, indicating that (1) poly-GR also inhibited protein translation in a length-dependent manner, (2) the inhibitory effect of (GR)20 is specific because amyloid β did not inhibit protein translation, (3) poly-PR is more potent in the inhibition of translation. Because there was a significant difference in the degree of translation inhibition between (PR)20 and (GR)20, we next compared their cytotoxicity. First, we treated NSC34 cells with FITC-conjugated DRPs and compare their penetration rate. As Fig. S3C shows, (PR)20 entered NSC34 cells more efficiently, but (GR)20 also entered the cells as well. Next, we treated NSC34 cells with (PR)20 or (GR)20 and measured their cytotoxicity. Consistent with the rate of translation inhibition as well as the efficiency of penetration, (PR)20 exerted stronger cytotoxicity (Fig. S3D).

Based on the results from IVT using poly-PR peptides with different length, even (PR)8 repeat might have cytotoxicity. However, when we challenged NSC34 cells with these poly-PR peptides with various lengths, minimal requirement of peptide repeat length for cytotoxicity was 16 and poly-PR peptides shorter than 16 had little toxicity even with high concentration (Fig. 5C). To explain the discrepancy of translation inhibition and cytotoxicity, we investigated the rate of cell penetration of these poly-PR variants because the cationic CPP poly-arginine has optimal length between 7–15 for efficient cellular uptake^16,20^ and thus short (PR) peptides might not satisfy the requirement for the cell penetration. To test whether the length of (PR) repeats influences the translocation of the peptides, we performed cell penetration assay using FITC-labeled (PR)12 and FITC-labeled (PR)20 with live or fixed/permeabilized HEK293 cells. When added to the culture medium of live HEK293 cells, FITC-(PR)20, but not FITC-(PR)12, penetrated the plasma membrane and accumulated to nucleoli (Fig. 5D). After the fixation/permeabilization, both of the peptides entered the cells and accumulated to nucleoli, indicating that the difference of cell toxicity between (PR)12 and (PR)20 was determined by the degree of cell penetration rather than the degree of inhibition of translation, because the level of translation inhibition by (PR)12 was comparable to the level by (PR)20 (Fig. 5B). It has been reported that HA-tagged (GR)20 peptide has a shorter half-life (less than 1 hr) in the culture medium and that may contribute to the lessor toxicity of poly-GR^4^. To deny the possibility that the lack of cell toxicity of shorter poly-PR peptides is due to their instability, we carried out a functional assay of (PR)12 after incubation in the culture medium. After incubation in the culture medium for 2 hr, (PR)12 still could accumulate to nucleoli of fixed/permeabilized HEK293 cells, showing that (PR)12 is stable in the culture medium (Fig. S4).

To investigate if the secondary structure of the poly-PR peptides might affect their efficiency of translocation, we monitored circular dichroism (CD) spectra of R12, (PR)12 and (PR)20 because artificially designed CPP containing (Arg-Arg-Pro) repeats preferably formed α-helix-like structure in cellular environment and thus had enhanced penetration rate^21^. Figure 5E,F showed CD spectra of R12, (PR)12 and (PR)20 in 20 mM Tris buffer (pH7.4) solution (Fig. 5E) and in 1.0 w/v% sodium dodecyl sulfate (SDS)-Tris buffer (pH7.4) solution (Fig. 5F), which mimicked amphipathic environments such as the plasma membrane. The spectra of all these peptides exhibited negative maxima at around 200 nm and weak negative maxima at 235 nm, suggesting these peptides basically formed random secondary structure under physiological condition. Mean residue molar ellipticity of (PR)12 and (PR20) were almost identical, indicating that both peptides had similar secondary structure. On the other hand, addition of SDS markedly influenced the secondary structure of poly-PR, resulting in the CD spectra with negative maxima at 210 nm and 230 nm, implying that poly-PR peptide formed a α-helix-like structure in amphipathic condition^21^. The effect of SDS on the structural change was more obvious in (PR)20 compared with (PR)12 (Fig. 5F).

**Figure 6:**
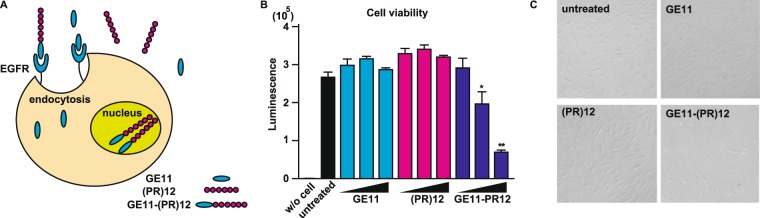
Enforced delivery of poly-(PR)12 rescued the cytotoxicity. (**A**) Scheme of GE11 peptide-mediated endocytotic delivery system. (**B**) Cytotoxicity of GE11 peptide, (PR)12 peptide or GE11-(PR)12 peptides were monitored by release of LDH. Human fibroblasts were treated with each peptide at 10 μM, 20 μM or 50 μM for 48 hr. N = 6. (**C**) Microscopic image of human fibroblasts treated with 50 μM of each peptide for 48 hr. Each experiment was independently repeated at least 3 times. Asterisks indicate a significant difference analyzed by one-way ANOVA followed by Dunnett’s test (**p < 0.01, *p < 0.05).

Based on these findings, we hypothesized that (PR)12 may cause cell death if it is successfully delivered into cells by a molecular cargo because it still possessed the inhibitory effect on protein translation (Fig. 5A). To examine this idea, we synthesized a (PR)12 peptide fused to GE11 peptide. GE11 sequence (YHWYGYTPQNVI: 12 a. a.) was found using a phage display screen to identify peptide sequences which have high affinity to epidermal growth factor receptor (EGFR)^22^. Despite its high affinity to EGFR (Kd = 22 nM), its mitogenic activity is much lower than EGF. When GE11 binds to EGFR, GE11-EGFR complex becomes internalized thus GE11 is a potential drug delivery carrier into the cells (Fig. 6A). To examine whether internalized (PR)12 by fused GE11 sequence exerts cytotoxicity, we treated human fibroblasts with GE11, (PR)12 or GE11-(PR)12 peptide and incubated the cells for 48 hr. (PR)12 fused to GE11, but not GE11 or (PR)12 alone, caused cell death, showing that although it lacks the ability to enter cells, (PR)12 still possesses the cytotoxic property (Fig. 6B,C).

## Discussion

Discovery of C9orf72 has opened a new horizon in the research field of ALS and FTD because it dominates the familial cases of these miserable disorders. From the standpoint of biochemistry and molecular biology, findings of RAN translation and associated arginine-containing dipeptides are of interests due to their intriguing characteristics including the unique translation system as well as cell penetration and cytotoxicity. Despite of the importance, little was known about the mechanism how Arg-rich dipeptides from C9orf72 penetrate the plasma membrane and cause cell death. In this study, we characterized the poly-PR and poly-GR peptides by comparing with other arginine-rich CPPs and found that only poly-PR and poly-GR, but not other Arg-rich CPPs inhibit protein translation, resulting in cell death. Extracellular pH affected the penetration rate of poly-PR and enforced delivery of non-penetrating short poly-PR peptide induced cytotoxicity, proposing a possible therapeutic target for C9orf72-mediated neurotoxicity.

CPPs have been extensively investigated because of their potential as a drug delivery tool. Especially cationic CPPs are attracting attentions because of their high efficiency of translocation and lower toxicity, and intracellular delivery of drugs, nucleotides and proteins by cationic CPPs have been achieved without significant cytotoxicity^23–25^. The key component of the cationic CPPs is arginine residue which has a strong positive charge. When compared with CPPs containing another positive amino acid lysine, CPPs containing Arg have higher efficiency to enter cells so that CPPs with high Arg contents attract more attentions^20^. The Arg content of (PR)20 (50%) and (GR)20 (50%) was comparable to those of other cationic CPPs, TAT (46%) and FHV (73%), and all of these peptides penetrated the plasma membrane and entered cells efficiently (Fig. 1B). However, (PR)12 which also has 50% of Arg content did not penetrate the plasma membrane, suggesting that not only the proportion of cationic amino acids but also other factors including the structure of peptide also regulate the efficiency. One such possible factor is formation of α-helix-like structure because artificially enhanced α-helix formation of cationic CPP results in better uptake rate^21^. Because the repeat length of PR regulated the penetration efficiency, we speculated (PR)20 might form more α-helix-like structure than (PR)12 did, and CD spectra under amphipathic condition showing that (PR)20 tended to form α-helix-like structure more than (PR)12 supported the idea (Fig. 5F).

Although cationic CPPs TAT, FHV and poly-PR/GR peptides, have been similarly reported to interact with anionic molecules including RNA^5,16,26^, their intracellular distributions were highly varied. R12 and FHV were diffusely localized to both cytosol and nucleus, but (PR)20 almost exclusively localized to the nucleus, especially nucleoli and (GR)20 localized to both nucleoli and cytosol. The difference of subcellular localization of poly-(PR) and poly-(GR) were also confirmed by ectopic expression of GFP-fused proteins. Because their Arg content was identical (50%), the difference was dependent on the nature of Pro and Gly inserted between Arg residues.

The specific distribution of poly-(PR) to the nucleolus was diminished by RNAse treatment after PFA fixation, indicating that RNA, but not RNA-binding proteins, was major binding partner of poly-(PR). Consistent with the aberrantly high affinity of poly-(PR) to RNA, cell toxicity assays and IVT assays showed that poly-PR had cytotoxicity through translation inhibition. Based on the findings that (PR)12, but not (GR)12 or R12, inhibited protein translation (Figs 3D, 5B and S3), not only the number of Arg residues, but also proline residue might play a role in determining the toxicity level. However, which aspect of the proline confers the toxic property to the poly-PR peptide still remains under investigation.

Pure Arg oligomer is a good model to investigate molecular mechanisms how these cationic CPPs penetrate plasma membrane. Several hypotheses such as the energy-dependent endocytotic model, energy-independent direct translocation model and cell surface receptor molecules-mediated internalization model, have been proposed^7^. As we demonstrated here, penetration rates of poly-PR as well as poly-Arg were controlled by the extracellular pH, and lowering temperature had little effect on the uptake, indicating that poly-PR entered cells independently from the energy-requiring endocytotic pathway. How the extracellular pH affects the penetrating rate remains elusive, but deprotonated fatty acids in the plasma membrane are supposed to attract the cationic polypeptide^19^.

It has been established that the length of Arg oligomer (n = between 7–15 is optimal) determines the efficiency of translocation and thus it is speculated that not only the positive charge which Arg residue harbors, but also structural factors limit the efficiency^16^. Consistent with this observation, the repeat number of poly-PR also controlled the penetrating rate and thus determined cellular toxicity. The (PR)12 peptide, which had the inhibitory effect on protein translation in a cell free system, did not show cytotoxicity because it could not enter cells. When fused to GE11 cargo, however, it was internalized by the endocytotic pathway and exerted its cytotoxicity, indicating that inhibition of uptake of the PR peptides might block neurotoxicity and suppress progression of diseases. Although the physiological significance remains to be determined, DRPs from C9orf72 actually transmit from cells to cells in neighborhood via the exosome-dependent and exosome-independent pathways^27,28^, and even are secreted to cerebrospinal fluid of ALS patients^29^, suggesting that intercellular communication via DRPs may contribute to the development of diseases and inhibition of uptake of DRPs could be a novel therapeutic target. One possible approach against extracellular DRPs is the antibody-mediated immunotherapy and it has been shown that the anti-GA antibody prevents the transmission of poly-GA and blocks its seeding activity, suggesting that anti-PR antibody may also prevent the penetration of poly-PR^28^.

As we reported previously, we proposed the neurotoxicity model of DRPs via inhibition of protein translation, and recently Zhang *et al*. reported that poly-GR impairs protein translation *in vivo* and causes neuronal loss in a mouse model of C9orf72-ALS, supporting our hypothesis^30^. In addition to our theory, a number of hypotheses such as aberrant ER stress, deficit in nucleocytoplasmic transport and inhibition of membrane-less organelles have been proposed. It is also reported that poly-(GR) exerts its toxicity through mitochondrial oxidative stress and DNA damages^31^. As we demonstrated here, structural factors, such as the length of dipeptides and existence of glycine residues or proline residues, determined the degree of translation inhibition, and thus it is also possible that these factors may be the determinant of the toxicity through other mechanisms.

Taken together, our study clarified the essential components for cell penetration and cytotoxicity by poly-PR/GR peptides, and modulation of extracellular environment which can mitigate the uptake and thus toxicity of the peptide might be a novel option to prevent motor neuronal death in ALS and FTD.

## Materials and Methods

### Antibodies and reagents

Anti-B23/nucleophosmin antibody was purchased from Cell signaling technology (Danvers, MA). RNAse A was obtained from Qiagen (Hilden, Germany). 1-Step Human Coupled IVT kit, oleic acid, poly-A RNA, Prolong Gold with DAPI and Nucblue live reagent were purchased from ThermoFisher Scientific (Waltham, MA). Cytotoxicity LDH Assay Kit-WST and octanol were obtained from Wako pure chemicals (Japan). Cell Titer-Glo and Cell-tox glo kits were purchased from Promega (Madison, WI). TAMRA-labeled random 15mer RNA was synthesized by Fasmac (Japan). Anti-puromycin antibody was purchased from Millipore.

### Peptides

FITC-labeled recombinant human insulin (51 a.a.) was purchased from Sigma. Amyloid beta-40 (40 a.a.) was obtained from Peptide Institute (Osaka, Japan). CPPs and C9orf72-Arg-rich dipeptides were synthesized by Genscript (Piscataway, NJ). The purity of the peptides was at least 85%. After synthesis, trifluoroacetate was replaced by acetate.

### LDH assay

LDH assay was performed with Cytotoxicity LDH Assay Kit-WST from Wako (Japan) following the manufacture’s protocol. Briefly, mouse neuronal NSC34 cells or human fibroblasts were plated on a 96-well plate at 2 × 10^4^ cells/well and then treated with the indicated concentration of peptide for 24 hr (NSC34 cells) or 48 hr (fibroblasts) before measurement of LDH release. The absorbance was measured by iMark microplate absorbance reader (Biorad).

### Buffers

All buffers used for the peptide absorption assay were prepared with 140 mM NaCl, 2.5 mM KCl, 5 mM HEPES, 5 mM glycine, and the final pH was adjusted with NaOH or HCl.

### Peptides absorption assay

The assay to show the effect of pH in peptides absorption into a hydrophobic phase was performed as previously reported^19^. FITC-labeled R12 or (PR)20 peptides were diluted to a final concentration of 10 μM in each buffer. Fifty microliter of the buffer-peptide mix and 50 μl of octanol containing 1% of oleic acid (OA) (v/v) were placed into a microtubes and vortexed for 5 min, followed by centrifugation at 2200 g briefly to separate the octanol phase and buffer phase. After separation, octanol phase containing OA and absorbed peptides were collected and the fluorescence was measured by a plate reader TECAN infinite F200 (Mannedorf, Switzerland).

### Peptide penetration assay

For imaging, Hela cells were washed twice with phosphate buffered saline (PBS) (pH7.4) and then incubated with PBS of different pH (pH6.0, pH7.4 and pH8.0) containing 2 μM of FITC-(PR)20 or FITC-R12 for 1 hr, followed by washing with PBS twice. For quantification, HEK293 cells were incubated with 10 μM FITC-(PR)20 in 10% FBS-DMEM for 30 min at 4 °C or 37 °C. The cells were washed twice with PBS and then re-suspended in PBS. The fluorescent signal was measured by a plate reader TECAN infinite F200.

### Crosslinking assay

HA-tagged (PR)20 was mixed with 10 mM of adenine, adenosine, ribose or ribose-3′ phosphate in the presence or absence of 10 μM poly-A and incubated for 5 min at room temperature. Crosslink was performed with 5 mM DSP (Pierce) for 30 min. The crosslinked peptides were then applied to SDS-PAGE followed by Coomassie blue staining.

### *In vitro* translation (IVT) assay

IVT assay was performed with 1-step Human-Coupled IVT kit (ThermoFisher) following manufacturer’s protocol. Briefly, turboGFP cDNA and Hela cell lysates were mixed in a 96 white-well PCR plate in the presence or absence of 100 μM of each peptide and incubated at 37 °C. The fluorescence from translated turboGFP was measured by LightCycler (Roche lifescience) every 1 min.

### Imaging of live HEK293 cells treated with fluorescent peptides

HEK293 cells were treated with 10 μM of FITC-labeled CPPs for 1 hr in combination with Nucblue live dye and then washed with PBS twice. The images were obtained by FV10 confocal microscopy (Olympus, Japan) or LSM710 confocal microscopy (Zeiss, Germany).

Imaging of fixed HEK293 cells treated with fluorescent peptides in combination with RNAse A treatment.

For RNAse A treatment, the HEK293 cells, fixed and permeabilized, were incubated with 10 μg/ml of recombinant RNAse A for 1 hr at 37 °C, followed by treatment with 10 μM of FITC-labeled peptides for 30 min. The cells were then washed with PBS twice and imaged with a confocal microscopy.

### Immunostaining of fixed HEK293 treated with fluorescent peptides

HEK293 cells were pretreated with 10 μM of FITC-labeled peptides for 2 hr and fixed with 4% PFA. After fixation, the cells were permeabilized with 0.1% Triton-X100 followed by incubation with 0.1% bovine serum albumin-PBS for blocking. The cells were immunostained with anti-B23 primary antibody and visualized by Cy3 labeled anti-rabbit secondary antibody.

### Colocalization analysis

Colocalization of signals from FITC-(PR)20 and DAPI were analyzed by ImageJ and Coloc2 plugin. The slope reflects the rate of association.

### CD spectra

CD spectra of peptides were measured by J-820 CD spectrometer (JASCO, Japan) following manufacturer’s protocol. The cell length was 1 mm and 100 μM of peptide in buffer was measured at room temperature. The spectra shown were the average of eight measurements for each sample and signals from buffer were subtracted.

## Electronic supplementary material

Supplementary information

## Data Availability

All materials, data and associated protocols are promptly available to readers without undue qualifications in material transfer agreements.
