# Supplementary material for: Characterization of membrane penetration and cytotoxicity of C9orf72-encoding arginine-rich dipeptides
Source: Sci Rep. 2018 Aug 24;8:12740. doi: 10.1038/s41598-018-31096-z (PMC6109075; doi:10.1038/s41598-018-31096-z)

Supplementary Information

Characterization of membrane penetration and cytotoxicity of C9orf72-encoding arginine-rich dipeptides.

Kohsuke Kanekura[1¶], Yuichiro Harada[1], Mao Fujimoto[2], Takuya Yagi[3], Yuhei Hayamizu[4], Kentaro Nagaoka[5], and Masahiko Kuroda[1]

[1]Department of Molecular Pathology, Tokyo Medical University, Tokyo, Japan.

[2]School of Life Sciences, Tokyo University of Pharmacy and Life Sciences, Tokyo, Japan.

[3]Department of Neurology, KEIO University, Tokyo, Japan.

[4]Department of Organic and Polymeric Materials, Tokyo Institute of Technology, Tokyo, Japan.

[5]Laboratory of Veterinary Physiology, Department of Veterinary Medicine, Tokyo University of Agriculture and Technology, Tokyo, Japan

[¶]***Corresponding author:***
Kohsuke Kanekura, M.D., Ph.D.
Department of Molecular Pathology
Tokyo Medical University
Email: kanekura@tokyo-med.ac.jp

Figure Legends

Supplementary Fig. 1.

(A) Microscopic images of phase-separated RNA by (PR)20 peptide. (PR)20 peptide and poly-A RNA was mixed and incubated for overnight on a slide glass. The scale bar shows 20 μm. (B) The circularity and roundness of phase-separated RNA by (PR)20 peptide was calculated by ImageJ. The scale bar shows 20 μm. (C) The FRAP assay performed with HEK293 cells treated with FITC-(PR)20 peptide for 1hr. The white arrow indicates the area of photobleaching. (D) The FRAP recovery rate of HEK293 cells treated with FITC-(PR)20 peptide for 1hr (blue) or 24 hr (orange). N=3 for each condition. Error bars show S.D.

Supplementary Fig. 2.

(A) Immunocytochemistry of Hela cells overexpressing GFP-(PR)50 or GFP-(GR)50, treated with puromycin (2μg/ml for 1 hr). Newly synthesized proteins were detected by anti-puromycin antibody. (B) Peptides absorption assay using FITC-R12 or FITC-insulin. The arrows indicate the octanol phase and arrowheads show the aqueous buffer phase.

Supplementary Fig. 3.

(A) Fluorescence intensity of translated tGFP in the IVT assay treated with 100 μM of each peptide. N=3. (B) Fluorescence intensity of translated tGFP in the IVT assay treated with 100 μM of each peptide. N=3. (C) The fluorescence intensity from translocated FITC-(PR)20 and FITC-(GR)20. (D) Cytotoxicity of (PR)20 and (GR)20 were monitored by release of LDH. NSC34 cells were treated with each DRP for 24 hr. N=6. Error bars show S.D. Asterisks indicate a significant difference analyzed by one-way ANOVA followed by Dunnett's test (**: $p<0.01$, *: $p<0.05$).

Supplementary Fig. 4.

Confocal imaging of HEK293 cells treated with FITC-(PR)12 peptides. (Left panels) Live HEK293 cells treated with 20 μM (PR)12-FITC for 2hr. (Middle panels) The conditioned medium of the live HEK293 cells shown left was transferred to HEK293

cells fixed/permeabilized. (Right panels) HEK293 cells fixed/permeabilized were treated with DMEM-10%FBS medium containing freshly added 20 μM (PR)12-FITC.

Supplementary Fig. 5.

(A) Raw data of CD spectra of R12, (PR)12 and (PR)20 in Tris-buffer (pH7.4). (B) Raw data of CD spectra of R12, (PR)12 and (PR)20 in 1v/w% SDS-Tris buffer (pH7.4).

Supplementary Fig. 6.

The entire image of agarose gel electrophoresis shown in Fig. 2F.

Supplementary Figure 1

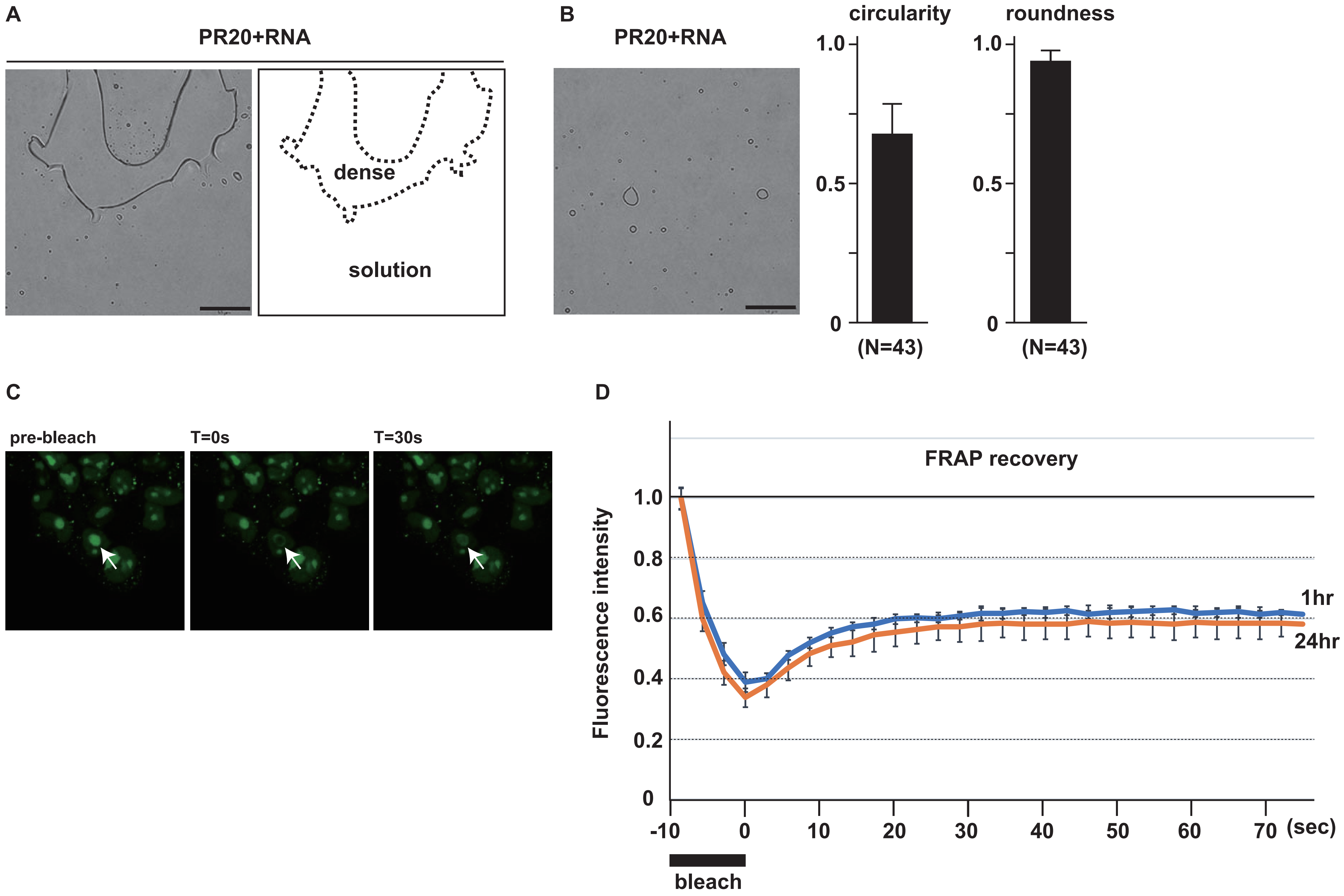

Supplementary Figure 2

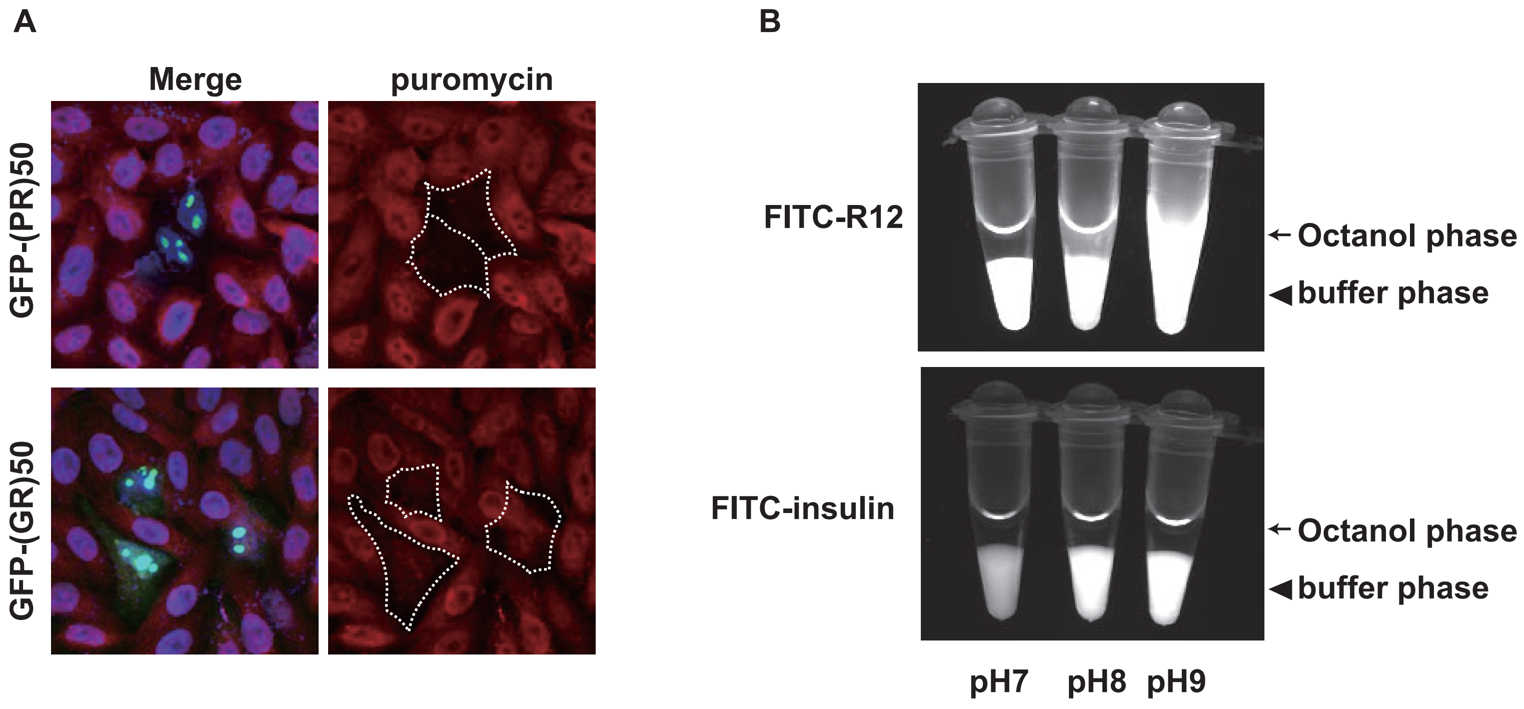

Supplementary figure 3

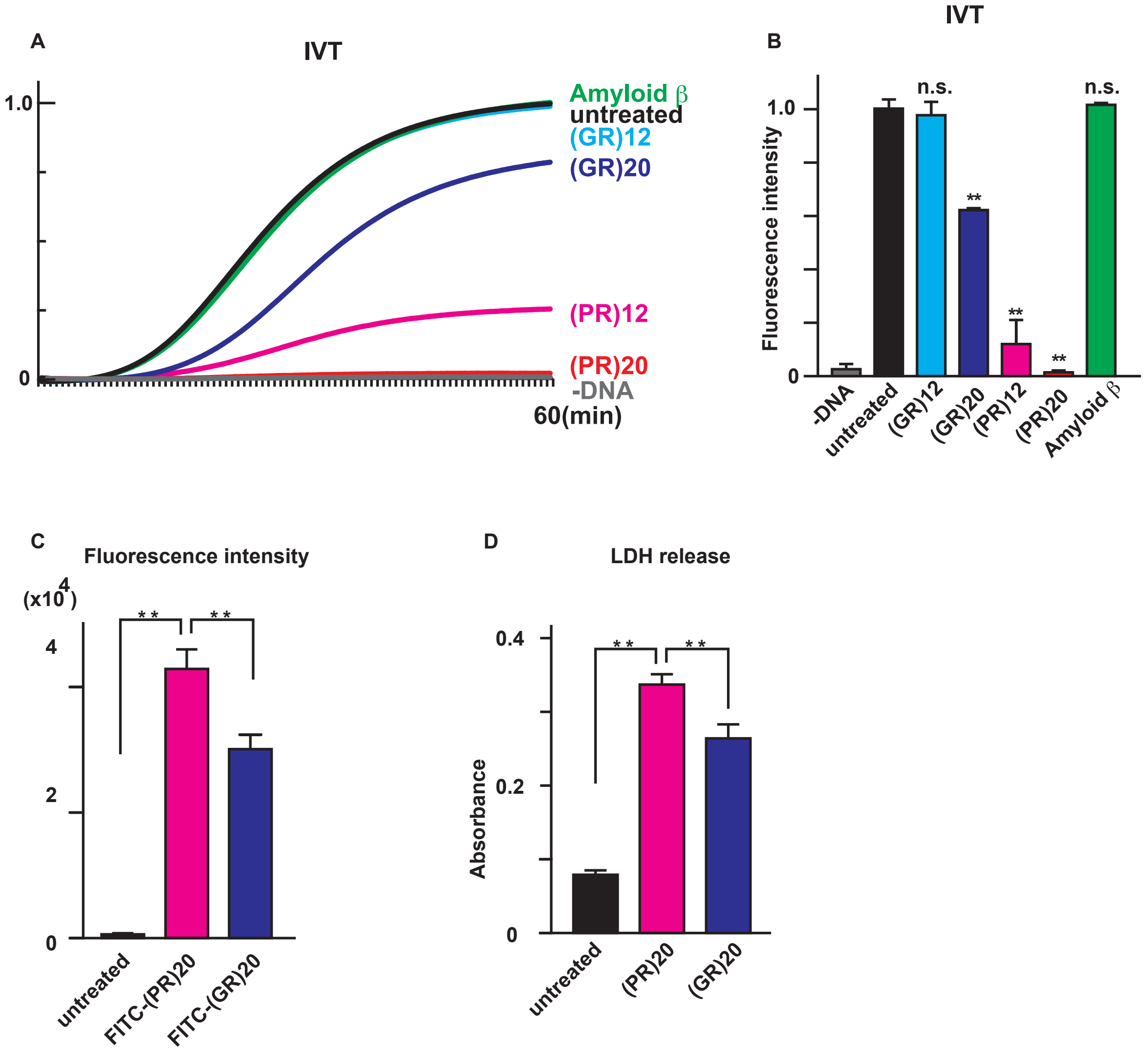

Supplementary figure 4

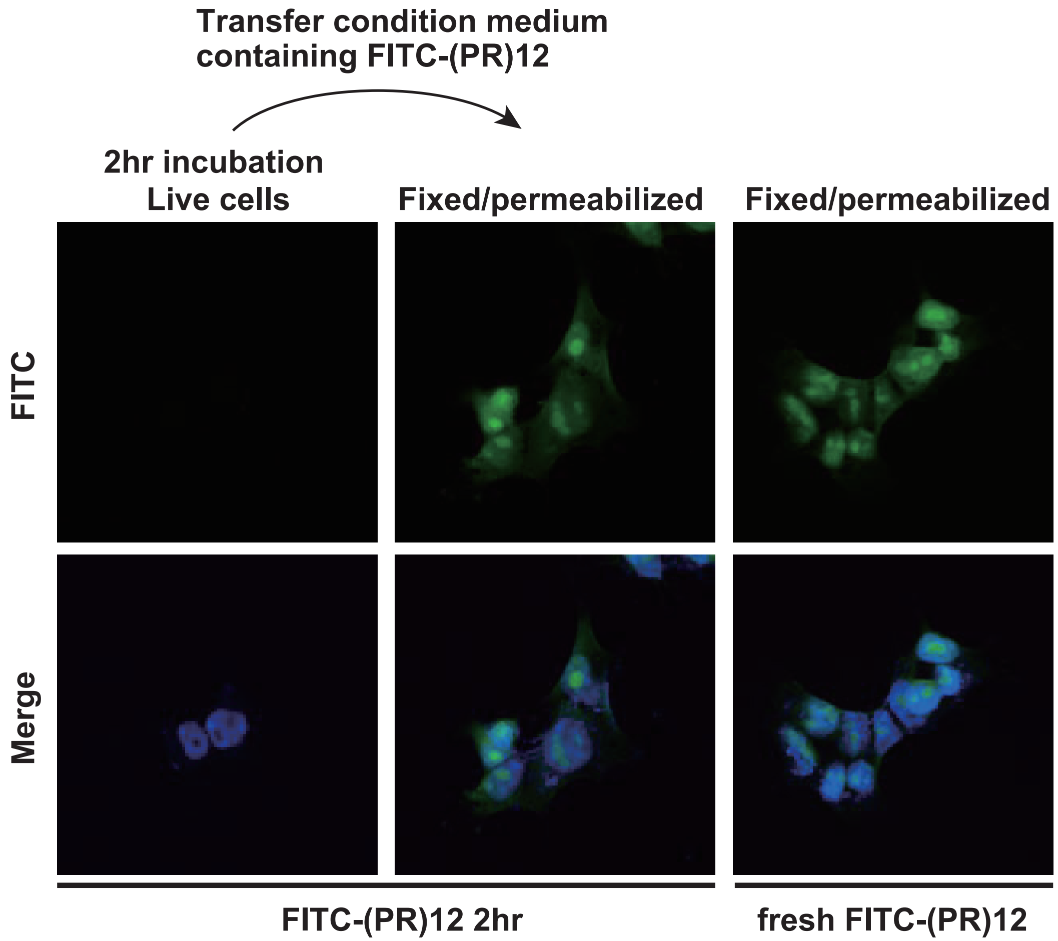

Supplementary Figure 5

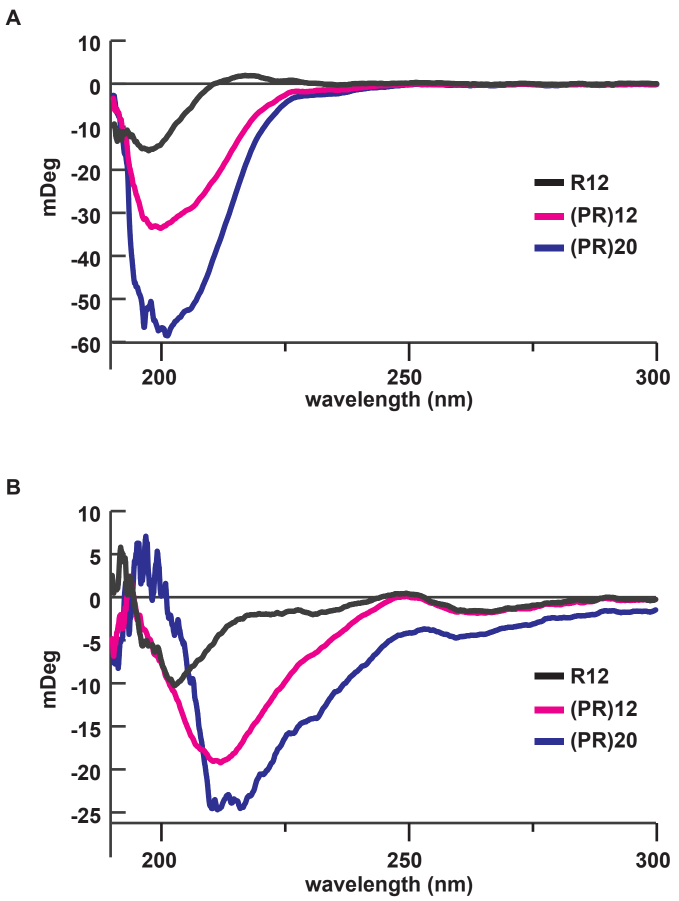

Supplementary Figure 6

A

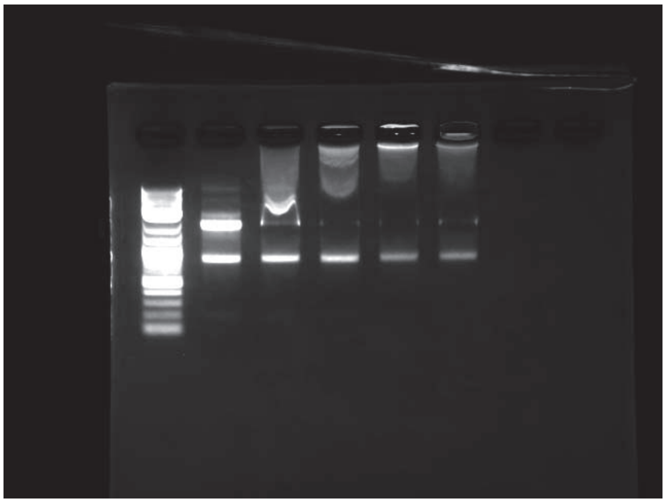

Supplement: Supplementary file 1 — Supplementary information [file 41598_2018_31096_MOESM1_ESM.pdf]
